# Supplementary material for: Determining the hierarchical order by which intestinal tract, administered diet, and individual relay can shape the gut microbiome of fattening quails
Source: PLoS One. 2024 Mar 21;19(3):e0298321. doi: 10.1371/journal.pone.0298321 (PMC10956773; doi:10.1371/journal.pone.0298321)
Supplement: S1 File — (DOCX) [file pone.0298321.s001.docx]

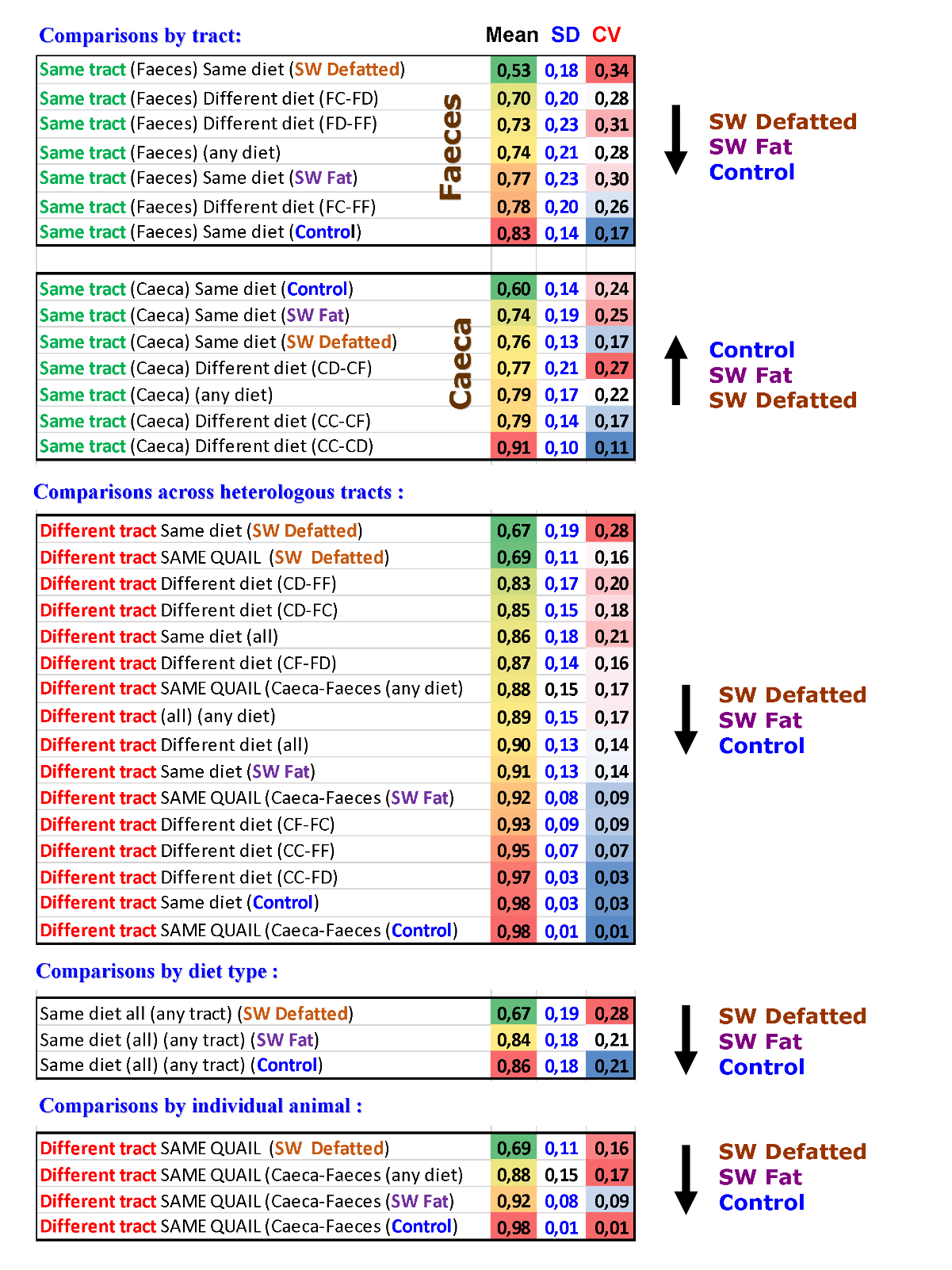


**Fig. S1, Rearrangement of Fig. 4 by splitting data in blocks grouped by each variable.** Data, from top to bottom, encompass: tract type in each of the two compartments, heterologous comparisons across the two tracts, diet type regardless of tract location, and, finally, comparisons restricted to caeca-faeces within the same animal, respectively. Each of the five panels’ table is ranked vertically by increasing order of Bray-Curtis community distance mean. On the right to each panel the order by which the three different diets resulted ranked is reported, along with fixed directional arrows (oriented in the SW Deafatted > SW fat > Control order) that allow to follow the compliance or the reversal of such order in each subset of the variables combinations.

**
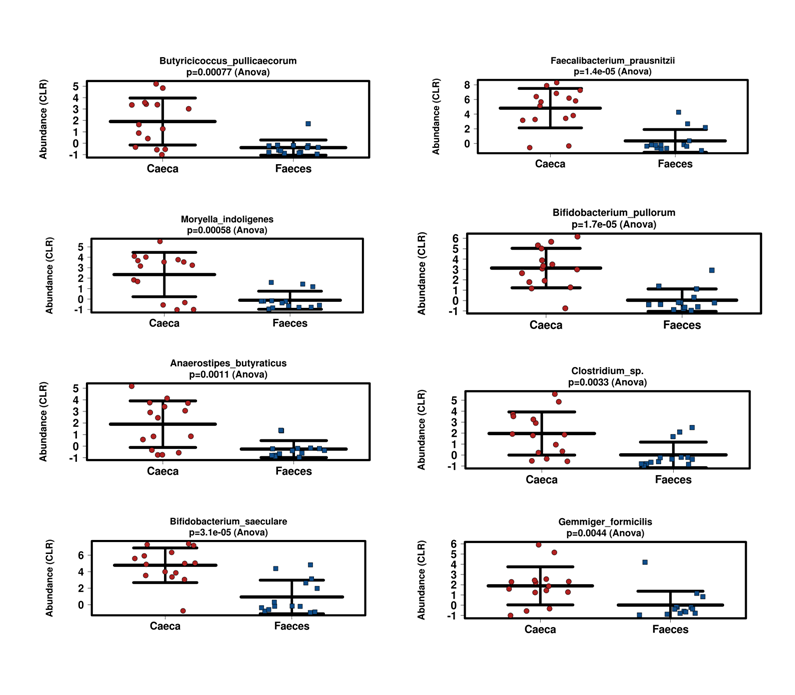
**

**Fig. S2. Details on cases which are significantly decreasing (p values shown) in Faeces in comparison to Caeca**

**
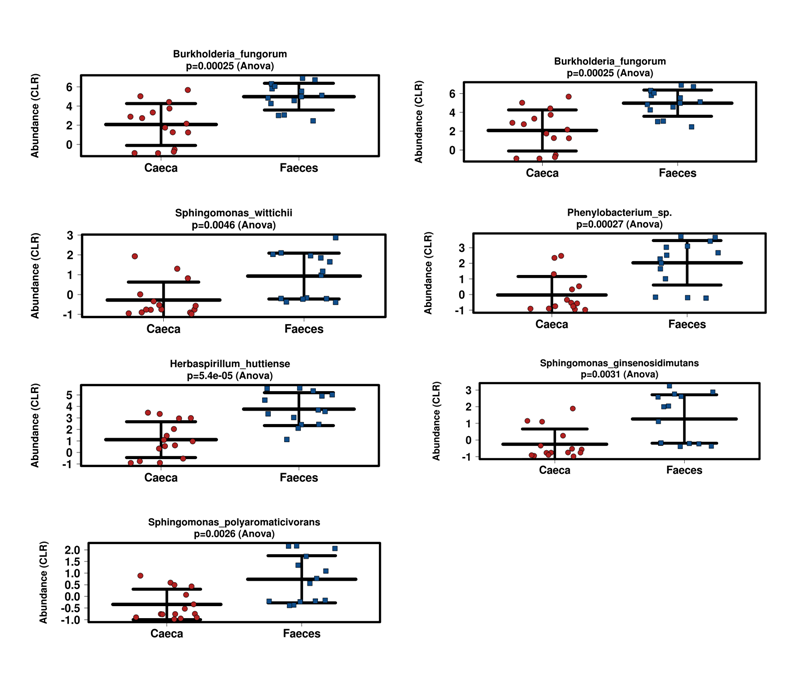
**

**Fig S3** **Details on cases which are significantly increasing (p values shown) in Faeces in comparison to Caeca**

**
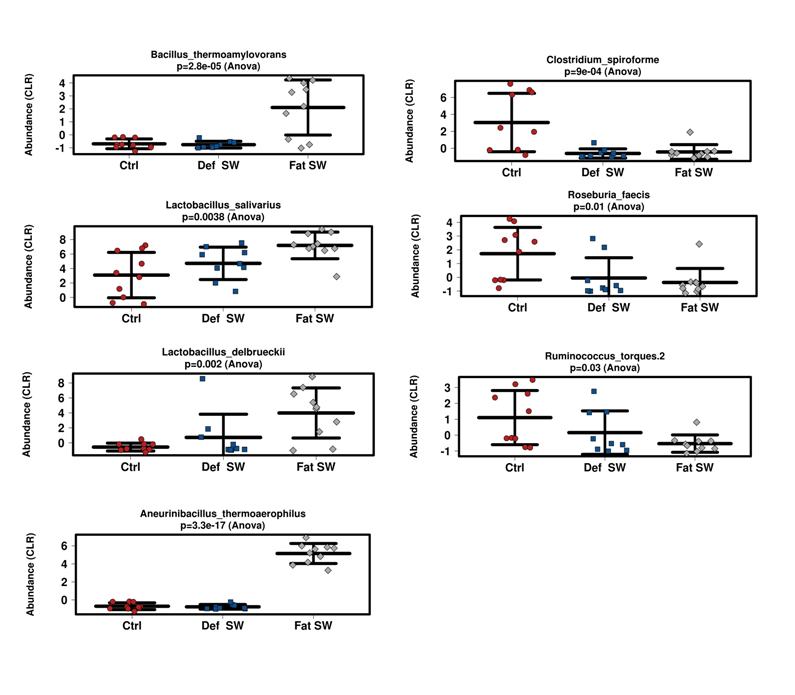
**

**Fig S4** **Details on cases which are significantly increasing (left panels) or decreasing (right panels)**

**(p values shown) in the SW diets**

**
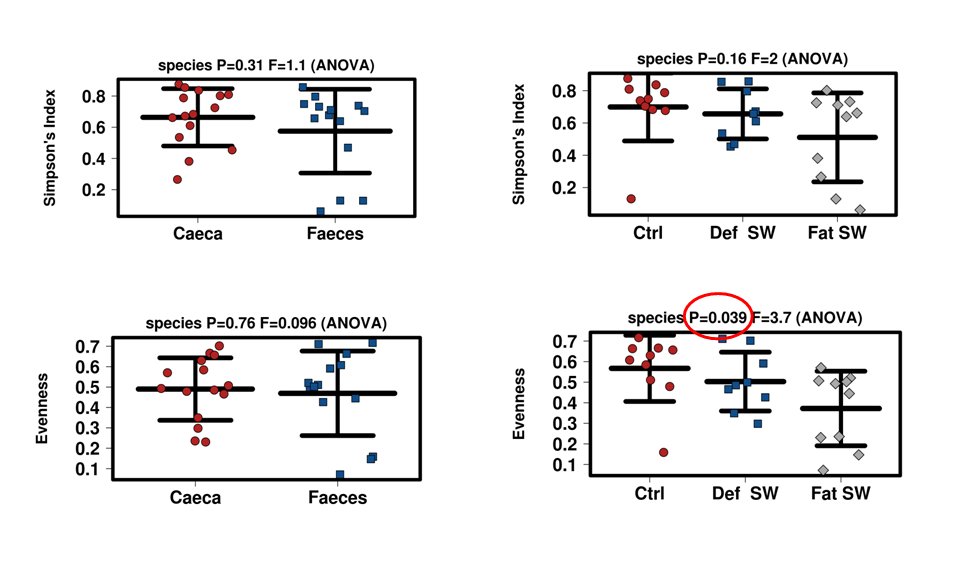
**

**Fig S5 Ecological indexes of Simpson’s diversity and community evenness comparisons in relation to gut tract /left panels) or diet type (right panels).** A statistically significant p value is evidenced by the red ellipsis.
